# Supplementary material for: Mitochondrial depolarization stabilizes the vitamin B12 chaperone MMADHC in the cytosol to increase MTR activity
Source: bioRxiv. 2025 Dec 31:2025.12.31.697091. Preprint. [Version 1] doi: 10.64898/2025.12.31.697091 (PMC12776266; doi:10.64898/2025.12.31.697091)
Supplement: 1 [file NIHPP2025.12.31.697091V1-supplement-1.pdf]

bioRxiv preprint doi: https://doi.org/10.64898/2025.12.31.697091; this version posted December 31, 2025. The copyright holder for this preprint (which was not certified by peer review) is the author/funder, who has granted bioRxiv a license to display the preprint in perpetuity. It is made available under aCC-BY 4.0 International license.

S1.

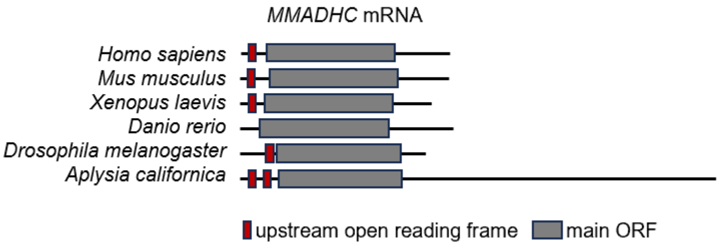

Fig. S1. Upstream open reading frames in MMADHC mRNA, related to Figure 1. Schematic of upstream open reading frames (uORFs) in *MMADHC* mRNA in the indicated species. The mRNA and main ORF lengths are drawn to scale, red boxes indicate presence of uORF.

bioRxiv preprint doi: https://doi.org/10.64898/2025.12.31.697091; this version posted December 31, 2025. The copyright holder for this preprint (which was not certified by peer review) is the author/funder, who has granted bioRxiv a license to display the preprint in perpetuity. It is made available under aCC-BY 4.0 International license.

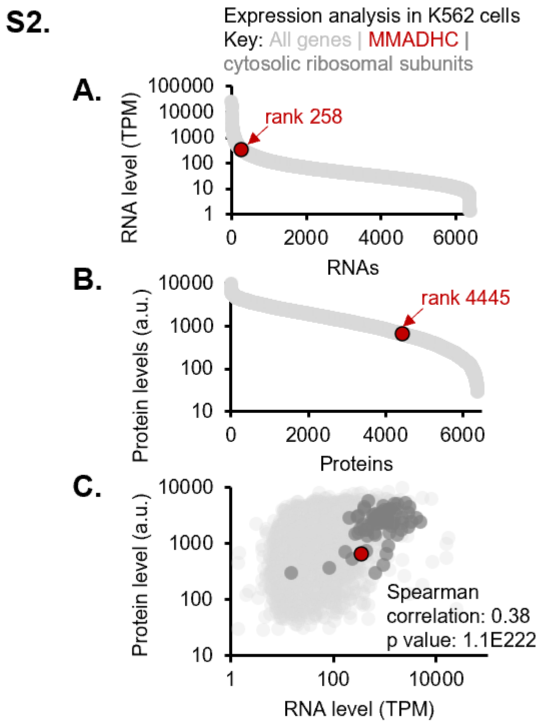

Fig. S2. MMADHC protein, but not RNA, is lowly expressed, related to Figure 2. (a) Genes ranked by RNA level in naïve K562 cells measured by RNAseq. (b) Genes ranked by protein level in naïve K562 cells measured by TMT proteomics. Bottom: Scatter plot of protein vs RNA level. Red point corresponds to MMADHC.

bioRxiv preprint doi: https://doi.org/10.64898/2025.12.31.697091; this version posted December 31, 2025. The copyright holder for this preprint (which was not certified by peer review) is the author/funder, who has granted bioRxiv a license to display the preprint in perpetuity. It is made available under aCC-BY 4.0 International license.

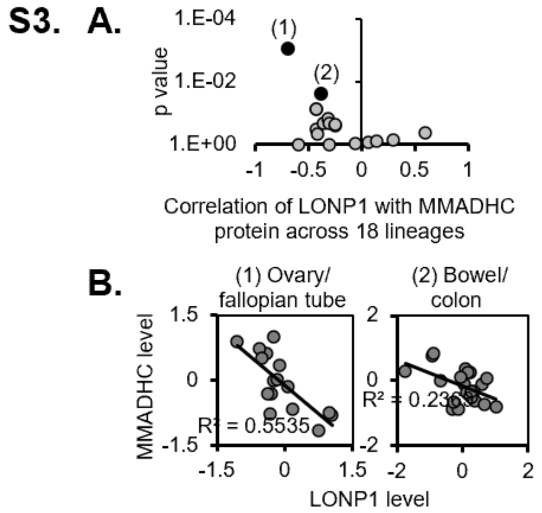

Fig. S3. Correlation of MMADHC and LONP1 across CCLE by lineage, related to Figure 2. (A) Volcano plot of correlation between MMADHC and LONP1 in each CCLE lineage. (B) Scatter plots of MMADHC vs LONP1 protein levels in the top two lineages in which the two proteins are most significantly negatively correlated.

bioRxiv preprint doi: https://doi.org/10.64898/2025.12.31.697091; this version posted December 31, 2025. The copyright holder for this preprint (which was not certified by peer review) is the author/funder, who has granted bioRxiv a license to display the preprint in perpetuity. It is made available under aCC-BY 4.0 International license.

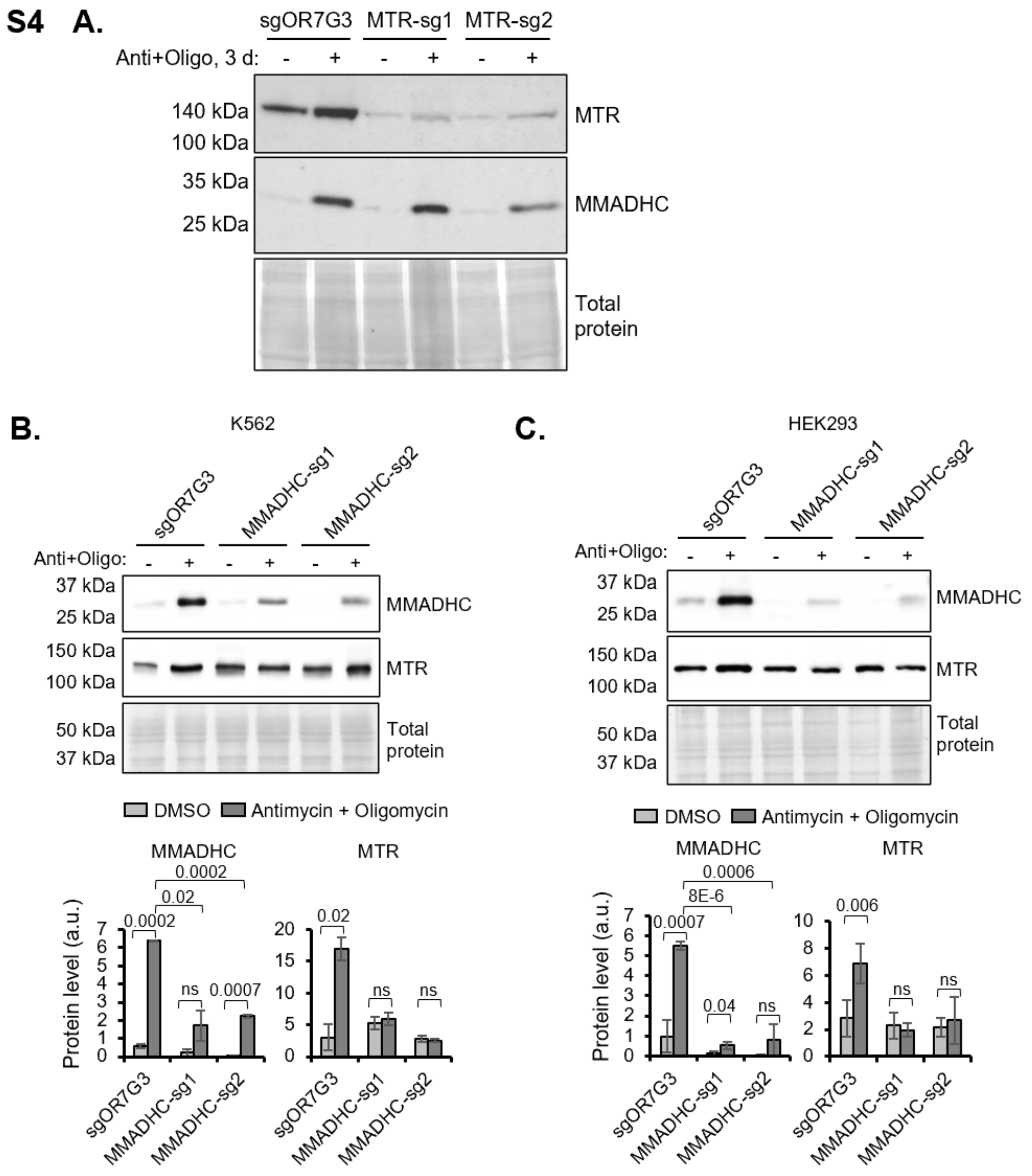

Fig. S4. Increase of MTR upon mitochondrial depolarization is MMADHC-dependent and not vice versa, related to Figure 3. (A) Western blot analysis of MMADHC and MTR in cutting control (sgOR7G3) and MTR KO K562 cells treated with antimycin and oligomycin for 3 days. (B, C) Western blot analysis of MMADHC and MTR in cutting control (sgOR7G3) and MMADHC KO K562 (B) and HEK293 (C) cells treated with antimycin and oligomycin for 3 days. Bar plots show quantification of western blots normalized to total protein. Error bars show standard deviation across two to four biological replicates.

bioRxiv preprint doi: https://doi.org/10.64898/2025.12.31.697091; this version posted December 31, 2025. The copyright holder for this preprint (which was not certified by peer review) is the author/funder, who has granted bioRxiv a license to display the preprint in perpetuity. It is made available under aCC-BY 4.0 International license.

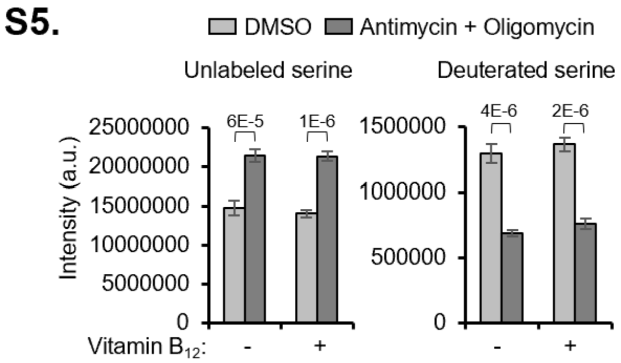

Fig. S5. Formate-to-serine flux decreases in cells treated with antimycin and oligomycin, related to Figure 3. Levels of undeuterated and deuterated serine quantified by mass spectrometry in K562 cells treated with antimycin and oligomycin for 3 days and labelled with fully deuterated formate for 9 hours in the presence or absence of vitamin B_{12}. Error bars represent standard deviation of four biological replicates.
